# Skin Rash Management Based on Early Elevation in the Eosinophil Proportion Within 3 Months of Initiating Apalutamide for Prostate Cancer

**DOI:** 10.1002/cnr2.70662

**Published:** 2026-08-23

**Authors:** Dai Koguchi, Hideyasu Tsumura, Ken‐ichi Tabata, Takefumi Satoh, Takashi Tachibana, Shuhei Hirano, Masaomi Ikeda, Daisuke Ishii, Kazumasa Matsumoto

**Affiliations:** ^1^ Department of Urology Kitasato University School of Medicine Sagamihara Kanagawa Japan; ^2^ Department of Urology Kitasato Institute Hospital Tokyo Japan; ^3^ Department of Urology Kitasato University Medical Center Kitamoto Saitama Japan

**Keywords:** apalutamide, prostate cancer, skin rash

## Abstract

**Background:**

Skin rash (SR) is a key issue in the long‐term use of apalutamide (APA) in patients with prostate cancer (PCa); however, there is currently no established strategy for the safe management of APA in preventing SR.

**Aims:**

To investigate the predictive value of early elevation in the proportion of eosinophils (EE) for SR in patients treated with APA for PCa.

**Methods and Results:**

We retrospectively reviewed the clinical data of 78 patients who received APA for PCa between April 2021 and November 2024. Early EE was defined as beyond the upper normal limit of 5.0% within 3 months of APA treatment initiation. The initial SR (I‐SR) and recurrent (R‐) SR incidences were compared between patients with and without early EE. During a median follow‐up period of 14.7 months, 35 patients (44.9%) developed SR, and a significantly higher proportion of patients with early EE had I‐SR than those without early EE (87.5% [7/8] vs. 40.0% [28/70]; *p* = 0.020). Six of the seven patients with early EE and I‐SR had EE before the onset of I‐SR (85.7%). In terms of R‐SR, among patients who reduced the APA dose after I‐SR, R‐SR remained significantly more prevalent in patients with early EE compared with that in patients without early EE (75.0% [3/4] vs. 11.1% [2/18]; *p* = 0.016).

**Conclusion:**

The present study suggests that early EE has predictive value for I‐ and R‐SR in patients who receive APA for PCa. Further large‐scale studies are warranted to verify the clinical utility of early EE in patients with APA.

## 1 Introduction

Skin rash (SR) following apalutamide (APA) administration for advanced prostate cancer (PCa) remains a stubborn issue, especially among Asian patients. The Phase 3 TITAN study found that SR with APA was observed approximately twice as frequently in Japanese patients (53.6%) relative to the overall cohort (24.4%) [1]. Such a high SR incidence was also found in recent real‐world data on Chinese (50.0%) and Korean (40.0%) patients [2, 3]. Because a consistent effect of APA was found among the overall cohort and Asian subpopulation in the TITAN study, investigating predictive factors for SR will assist in exploiting the therapeutic potential of APA in Asian patients [4]. Currently, although there is no established strategy for the safety management of APA in preventing recurrence of SR (R‐SR) and deterioration of SR, recent evidence from immune checkpoint inhibitor therapy suggests that peripheral eosinophilia positively correlates with the risk of immune‐related adverse events across various malignancies [5]. This association is likely driven by the accumulation of interleukin‐5 following CD4^+^ T‐cell activation [6]. Supporting this immunological rationale, a mouse drug allergy model previously demonstrated that APA induces a significant proportional increase in T cells (CD4^+^ and CD8^+^) and B cells [7]. Despite these mechanistic insights, clinical data regarding the association between eosinophil dynamics and APA‐induced SR are scarce [8]. Here, we aimed to assess the significance of early elevation in the proportion of eosinophils (EE) within 3 months after APA initiation in patients with PCa. We also investigated the association between initial (I‐) SR severity and the time from I‐SR onset to APA discontinuation.

## 2 Methods

We retrospectively reviewed the clinical data of 78 patients who received APA for PCa between April 2021 and November 2024. All patients had eosinophil levels tested from blood samples, and EE was defined as an eosinophil proportion > 5.0% within 3 months of APA treatment initiation. The upper normal limit of 5.0% for eosinophil levels is widely accepted and also adopted at the three hospitals participating in this study [9]. Patients who had developed EE before the initiation of APA were not classified as early EE. I‐ and R‐SR incidences were compared between patients with early EE and those without early EE. Additionally, an association between I‐SR severity and the time from I‐SR onset to APA discontinuation was investigated. The SR severity was assessed using the Common Terminology Criteria for Adverse Events (version 5).

The following data on patient characteristics were collected from the patients' medical charts, including demographic data, Charlson Comorbidity Index [10], age at the initiation of APA, initial dose of APA, resumed dose of APA, timing of appearance of SR (either I‐ or R‐SR), history of allergy, concomitant medications excluding APA, and reasons for the discontinuation of APA.

Categorical patient characteristics were compared using a *χ* ^2^ test (or Fisher's exact test, if appropriate). Univariable logistic regression analysis was performed to evaluate the association between early EE and the incidence of I‐SR. Odds ratios with 95% confidence intervals were calculated. All statistical analyses were performed using Stata 14 for Windows. All *p*‐values were two‐sided, with *p* < 0.05 considered significant.

## 3 Results

The study cohort consisted of 69 patients with metastatic hormone‐sensitive PCa and nine with nonmetastatic castration‐resistant PCa, with a median age of 72 years. All the patients diagnosed with the nonmetastatic castration‐resistant PCa had a prior history of combined androgen blockade, consisting of androgen deprivation therapy plus a first‐generation anti‐androgen as the initial treatment during hormone sensitive phase.

During a median follow‐up period of 14.7 months, EE occurred in 13 patients. Using the mean value for the period from APA introduction to EE (90.1 days) as the cut‐off point, eight and five patients were classified as early (range: 15–89 days) and late (range: 154–596 days) EE, respectively.

Patient characteristics are shown in Table 1. Demographic data such as age and body mass index did not significantly differ between patients with early EE and those without early EE, with a median proportion of eosinophils of 1.8 (0.9–4.3) and 2.5 (0.8–8.1), respectively (*p* = 0.47). In terms of comorbidities, the proportion of patients with a Charlson Comorbidity Index ≥ 1 was 37.5% (*n* = 3) in the early EE group and 50.7% (*n* = 35) in the non‐early EE group (*p* = 0.50). None of the patients had a history of drug allergies through the interviews, and one patient had previously received treatment for eosinophilia associated with nontuberculous mycobacterial infection but was not receiving medication during the study period.

**TABLE 1:** Patients' characteristics at the initiation of apalutamide according to early elevation of the eosinophil proportion.

Variable, *N* (%)	Early EE (*n* = 8)	Without early EE (*n* = 70)	*p*
Age (years), median (range)	73 (56–81)	72 (70–83)	0.64
Body mass index (kg/m^2^), median (range)	23 (15–31)	24 (19–26)	0.51
ECOG performance‐status score, *n* (%)			
0	5 (62.5)	41 (58.6)	0.83
1	3 (37.5)	29 (41.4)	
2	0	0	
PSA (ng/mL), median (range)	120 (6–1483)	155 (5–4391)	0.22
≥ Gleason score 9, *n* (%)	4 (50.0)	33 (47.8)	0.88
≥ Clinical T3b, *n* (%)	2 (25.0)	34 (49.3)	0.21
Clinical N1, *n* (%)	3 (37.5)	31 (44.3)	0.71
Metastatic status, *n* (%)			
Lymph node metastasis	3 (37.5)	34 (49.3)	0.55
Bone metastasis	8 (100)	64 (91.4)	0.39
Visceral metastases	0	14 (20.3)	0.16
Nonmetastatic castration‐resistant prostate cancer	1 (12.5)	8 (11.4)	0.93
Eosinophil proportion (%), median (range)	1.8 (0.9–4.3)	2.5 (0.8–8.1)	0.47
≥ Charlson Comorbidity Index 1	3 (37.5)	35 (50.7)	0.50
Comorbidities			
Hypertension	3 (37.5)	29 (42.0)	
Hyperlipidemia	2 (25.0)	14 (20.3)	
Hyperuricemia	1 (12.5)	4 (5.8)	
Diabetes mellitus	2 (25.0)	12 (17.4)	
Myocardial infarction	1 (12.5)	3 (4.3)	
Angina pectoris	0 (0)	5 (7.2)	
Cerebral infarction	2 (25.0)	3 (4.3)	
Malignant tumor	1 (12.5)	10 (14.5)	
Chronic obstructive pulmonary disease	0	2 (2.9)	
Renal dysfunction (> serum creatinine of 3.0 mg/dL)^a^	0	0	
Gastric ulcer	0	1 (1.4)	
Nontuberculous mycobacterial infection	0	1 (1.4)	
Peripheral neuropathy	0	1 (1.4)	

^a^ The Charlson Comorbidity Index defines renal dysfunction as > serum creatinine of 3.0 mg/dL.

In terms of the incidence of I‐SR, 35 patients (44.9%) developed SR, with early EE associated with a significantly greater prevalence of I‐SR than non‐early EE (87.5% [7/8] vs. 40.0% [28/70]; *p* = 0.020) (Table 1). The median eosinophil proportion at I‐SR in the eight patients with early EE was 9.3% (range: 5.9%–32.0%), and EE before the onset of I‐SR occurred in six of the seven patients (median: 8.8%, range: 5.8%–15.5%). Compared with other EE patterns, I‐SR was significantly more prevalent in the early EE group (vs. late EE: 20.0% [1/5], *p* = 0.032; vs. EE before the initiation of APA: 33.3% [3/9], *p* = 0.049). The median time to I‐SR after the initiation of APA in the overall cohort and in patients with early EE were 57.5 days (range: 5–400 days) and 22 days (range: 15–89 days), respectively. The odds ratio for early EE was 8.5 (95% confidence interval: 0.99–73.3, *p* = 0.052).

**FIGURE 1:**
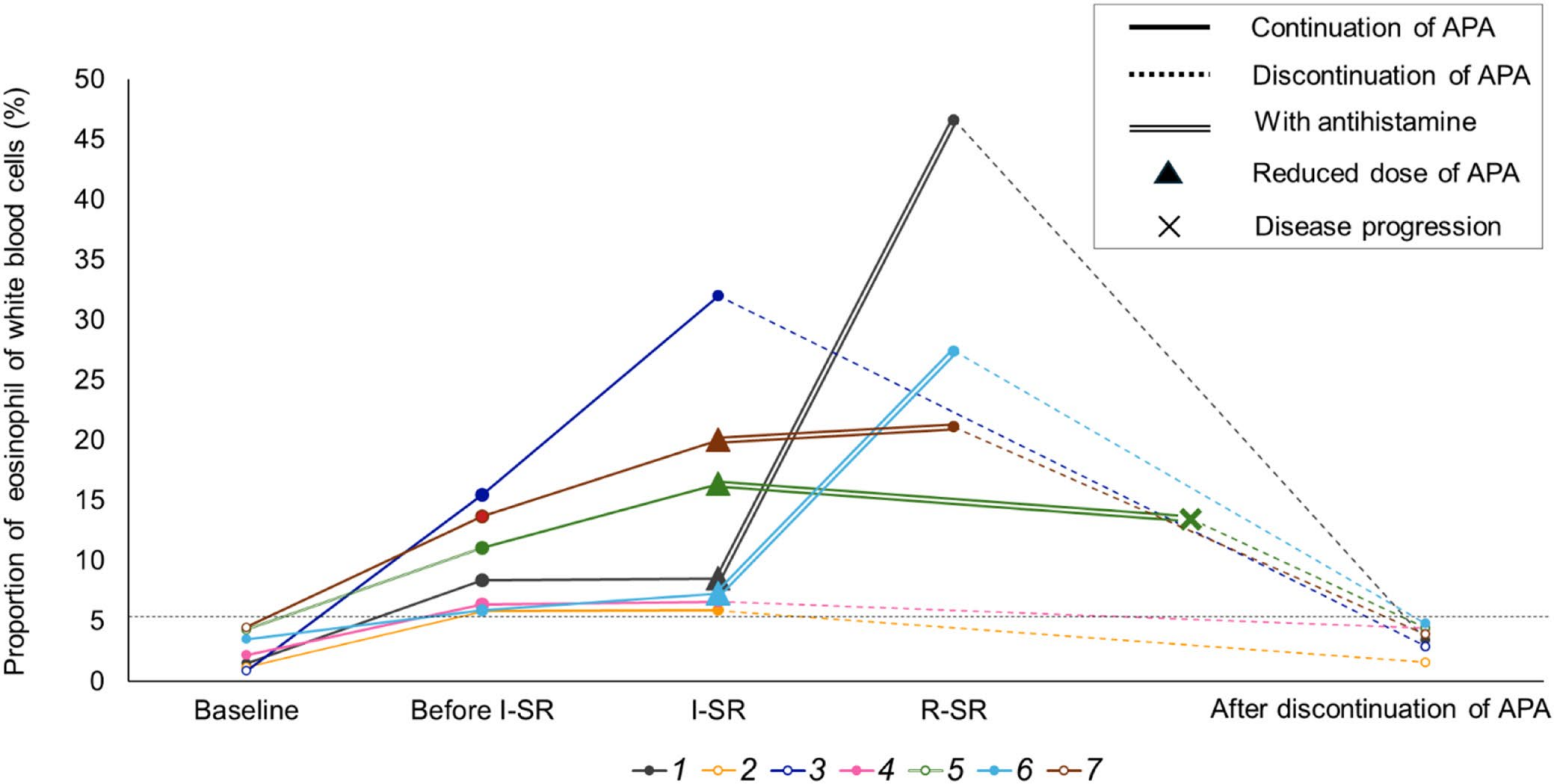
Chronological changes in the proportion of eosinophils among blood cells in patients with skin rash and early elevation of the eosinophil proportion within 3 months after apalutamide introduction.

After I‐SR, 25 patients were switched to a reduced APA dose, with five developing R‐SR during antihistamine treatment. Early EE was associated with a significantly greater incidence of R‐SR than non‐early EE (75.0% [3/4] vs. 11.1% [2/18]; *p* = 0.016) (Table 2). The proportion of eosinophils recovered to normal after APA treatment was discontinued in patients with I‐SR and early EE (Figure 1).

**TABLE 2:** Incidence of skin rash following apalutamide introduction according to early elevation of the eosinophil proportion.

	Early EE (*n* = 8)	Without early EE (*n* = 70)	*p*
Starting APA dose			
240 mg	8 (100)	58 (82.9)	0.44
180 mg	0 (0)	10 (14.3)	
120 mg	0 (0)	2 (2.9)	
Skin rash	7 (87.5)	28 (40.0)	0.020
Discontinuation after initial skin rash	3 (37.5)	3 (4.3)	0.013
Continuation of APA after initial skin rash	4 (50.0)	25 (34.3)	0.46
After initial skin rash			
Continuation of starting APA dose	0 (0)	4 (5.7)	0.99
Recurrence^a^	0 (0)	2 (50.0)	—
Switched to reduced APA dose	4 (50.0)	21 (30.0)	0.26
Recurrence^b^	3 (75.0)	2 (11.1)	0.016

*Note:* Values given as *n* (%).

Abbreviations: APA, apalutamide; EE, elevation of the eosinophil proportion.

^a^ Proportions were calculated based on the denominator of the number of patients who continued the starting APA dose after the initial skin rash.

^b^ Proportions were calculated based on the denominator of the number of patients who switched to a reduced APA dose after the initial skin rash.

When exploring a potential association between APA dose and SR, the prevalence of I‐SR was found to be significantly associated with a higher starting APA dose per body weight ratio in patients without early EE (> 3.7 mg/kg: 55.6% [15/27] vs. ≤ 3.7 mg/kg: 30.2% [13/43]; *p* = 0.035). This ratio displayed no significant predictive value in patients with early EE (> 3.7 mg/kg: 100% [2/2] vs. ≤ 3.7 mg/kg: 83.3% [5/6]; *p* = 0.54).

For the time from I‐SR onset to APA discontinuation, the proportion of patients with a period longer than 6 days was significantly greater in patients with Grade 3 SR (83.3%, 5/6) than in those with Grade 1 (14.3%, 1/7) or 2 SR (25%, 5/20) (*p* = 0.014). Early EE was not correlated with the SR severity (Grade 3: 25.0% [2/8]; Grade 2: 12.5% [1/8]; Grade 1: 50.0% [4/8]).

## 4 Discussion

The present study uniquely focused on early EE in patients receiving APA for PCa. We identified three findings relevant to the safety management of APA. First, patients with early EE were significantly more likely to have I‐SR than those without early EE, and approximately 85% of patients with early EE and I‐SR had developed EE before the onset of I‐SR. Second, R‐SR was significantly associated with early EE among patients who reduced the APA dose after I‐SR. Third, the period from I‐SR onset to APA discontinuation was positively correlated with the severity of I‐SR.

The present findings indicate that the mechanism of SR in PCa patients treated with APA can be roughly categorized as dose‐dependent or dose‐independent, with the latter potentially involving an allergic reaction, with eosinophilia classified as a Type IV hypersensitivity reaction [11]. In fact, the present study found that the R‐SR incidence was approximately seven times greater in patients with early EE than in those without early EE, even following APA dose reduction. Moreover, the eosinophil proportion in all patients with early EE returned to normal levels after discontinuing APA treatment. This potential predictive value of early EE for SR was supported by the results of a large prospective study using laboratory data for 3223 patients who were treated at a tertiary hospital, in whom Cox multivariable analysis revealed early EE as an independent risk factor for cutaneous and visceral reactions (hazard ratio, 10.49; 95% confidence interval: 3.13–35.16) [12]. Therefore, switching from APA to another agent could be considered in patients with early EE after I‐SR occurs. In patients without early EE, the results suggest that reducing the initial APA dose may be associated with a lower risk of R‐SR. Such a dose‐dependent mechanism of SR in patients without early EE is supported by our observed positive correlation between APA dose per body weight and the SR prevalence, consistent with recent Japanese data (cutoff: 3.3 mg/kg) [13]. However, as the present study was limited by a small sample size and the association between early EE and I‐SR was only borderline in the univariable logistic regression analysis (odds ratio, 8.5; 95% confidence interval, 0.99–73.3; *p* = 0.052), this EE‐based strategy for the management of SR should be validated in larger studies using multivariable logistic regression analyses, while also considering genetic data for predicting interindividual variability in the response to APA [14].

Additionally, the present study indicates a relationship between the time from I‐SR onset to APA discontinuation and SR severity. This may be because APA has a relatively long plasma half‐life of approximately 7 days [15], highlighting the importance of early recognition of I‐SR to prevent it from becoming severe.

This study had several limitations. First, the precise etiology of SR in these cases was histologically unclear because of a lack of biopsies to analyze. Second, because of the retrospective study design, the clinical outcomes may have been affected by residual confounding from other factors, despite the patients having no history of allergy or concomitant use of eosinophil‐affectable medicines, such as corticosteroids. Third, the cutoff points were defined based on the mean figures for the period from APA introduction to eosinophil elevation (90.1 days) and dose per body weight (3.7 mg/kg), not considering a specific established biologic threshold. Additionally, because we defined eosinophil elevation strictly by its proportion, these levels are subject to confounding by acute inflammatory states that alter other white blood cell populations, thereby limiting its interpretability as a standalone biomarker. Fourth, although early EE was associated with a markedly higher incidence of I‐SR, the association was only borderline in the logistic regression analysis. Given the limited sample size, these findings should be interpreted with caution and require confirmation in larger studies. Fifth, recall bias may have affected the reported timing of I‐SR onset, as the data were collected from patient interviews. Nevertheless, to the best of our knowledge, this is the first study to provide novel insights into the long‐term management of APA focusing on EE and the time from I‐SR onset to APA discontinuation.

In conclusion, the present study suggests that early EE has predictive value for I‐ and R‐SR in patients who receive APA for advanced PCa, indicating potential dose‐independent mechanisms of SR in early EE. Additionally, the periods from I‐SR onset to APA discontinuation may have a positive correlation with the severity of SR. Routine monitoring of eosinophil proportions may thus contribute to the early recognition of I‐SR in some patients treated with APA, helping to prevent the condition from becoming more severe. Moreover, this exploratory analysis suggests a potential strategy for future individualized management; while patients without early EE may have a lower risk of R‐SR following APA dose reduction, the emergence of early EE may be a factor in considering treatment discontinuation. Given the retrospective nature and small sample size of the present study, these findings should be considered hypothesis‐generating. Further large‐scale, prospective studies are required to verify the clinical utility of EE in the management of APA‐based treatment for PCa.

## Author Contributions

**Hideyasu Tsumura:** software, validation, investigation, data curation, visualization, writing – review and editing, formal analysis. **Masaomi Ikeda:** data curation. **Dai Koguchi:** conceptualization, methodology, software, investigation, data curation, visualization, writing – original draft, writing – review and editing, formal analysis, project administration. **Daisuke Ishii:** data curation. **Ken‐ichi Tabata:** validation. **Takashi Tachibana:** data curation. **Takefumi Satoh:** validation. **Shuhei Hirano:** data curation, investigation. **Kazumasa Matsumoto:** supervision.

## Funding

The authors have nothing to report.

## Ethics Statement

This study was approved by the Institutional Review Board of Kitasato University School of Medicine and Kitasato University Hospital (Kanagawa, Japan) (approval numbers B22‐102 and B24‐120). The study was conducted in accordance with the Declaration of Helsinki and its contemporary (2013) amendments.

## Consent

The Institutional Review Board waived the requirement for informed consent because of the retrospective nature of this observational study.

## Conflicts of Interest

The authors declare no conflicts of interest.

## Data Availability

The data that support the findings of this study are available on request from the corresponding author. The data are not publicly available due to privacy or ethical restrictions.
